# Role of sLOX‐1 in intracranial artery stenosis and in predicting long‐term prognosis of acute ischemic stroke

**DOI:** 10.1002/brb3.879

**Published:** 2017-12-13

**Authors:** Xian‐Mei Li, Ping‐Ping Jin, Jie Xue, Jie Chen, Qin‐Fen Chen, Xiao‐Qian Luan, Zeng‐Rui Zhang, Tie‐Er Yu, Zheng‐Yi Cai, Kai Zhao, Bei Shao

**Affiliations:** ^1^ Department of Neurology First Affiliated Hospital of Wenzhou Medical University Wenzhou China; ^2^ Department of Neurology Yangpu Hospital of Tongji University ShangHai China; ^3^ Department of Gastroenterology First Affiliated Hospital of Wenzhou Medical University Wenzhou China

**Keywords:** acute ischemic stroke, intracranial artery stenosis, long‐term functional outcome, Serum soluble lectin‐like oxidized low‐density lipoprotein receptor‐1

## Abstract

**Objective:**

The role of sLOX‐1 in acute ischemic stroke still remains unclear. This study aims to demonstrate the value of sLOX‐1 in evaluating degrees of intracranial artery stenosis and to predict prognosis in stroke.

**Methods:**

Two hundred and seventy‐two patients were included in this study and basic data were collected within 72 hr on admission. We assessed the association between sLOX‐1 levels and stroke conditions in one‐year duration. After adjusting for potential confounders, regression analyses were performed.

**Results:**

We found that sLOX‐1 levels were increased significantly in severe patients compared to the mild stroke group (*p *= .011). After adjusting confounders, sLOX‐1 was associated with a poor functional outcome in patients with an adjusted OR of 2. 946 (95% CI, 1.788–4.856, *p* < .001). There was also positive correlation between sLOX‐1 levels and the degrees of intracranial artery stenosis in the different groups (*p* = .029).

**Conclusions:**

Our study demonstrated that sLOX‐1 levels could be used to evaluate the severity of stroke and the degrees of intracranial artery stenosis. Furthermore, sLOX‐1 could be exploited to predict the long‐term functional outcome of stroke.

## 1 INTRODUCTION

Stroke is defined as focal neurological deficits caused by vascular rupture or obstruction of cerebral vessel in central nervous system. The consequence of stroke is regarded as the second most common cause of death after cancer and also the main cause of disability around the world (Huang et al., 2013). Ischemic stroke is the major subtype of cerebrovascular disease accounting for 80%‐85% of all strokes (Allen & Bayraktutan, 2009), with intracranial artery stenosis (IAS) considered to be a major causative factor worldwide (De Silva et al., 2007; Gorelick, Wong, Bae, & Pandey, 2008). IAS occurs when primary atherosclerosis ruptures, displacing the atheromatous plaque resulting in thrombotic occlusion of the intracranial arteries (Carvalho, Oliveira, Azevedo, & Bastos‐Leite, 2014).

Lectin‐like oxidized low‐density lipoprotein receptor‐1 (LOX‐1), the main receptor for Ox‐LDL in vascular endothelial cells (Sawamura et al., 1997), plays a crucial role in the pathogenesis of atherosclerosis (Mehta, Chen, Hermonat, Romeo, & Novelli, 2006). The expression of LOX‐1 is insignificant or undetectable in healthy vessels, but is comparatively over‐expressed in atherosclerotic lesions (Draude, Hrboticky, & Lorenz, 1999). LOX‐1 possesses an extracellular membrane‐bound protein with a single C terminal domain which can be cleaved and released as a soluble form (sLOX‐1) in the circulation system (Murase et al., 2000). The concentration of circulating soluble lectin‐like oxidized low‐density lipoprotein receptor‐1 (sLOX‐1), therefore, can be detected and serves as a reflection of LOX‐1 expression (Brinkley et al., 2008). Several reports have demonstrated that sLOX‐1 could be targeted as a potential biomarker of acute coronary syndrome (Hayashida et al., 2005) and other serious cardiovascular‐related diseases (Kobayashi et al., 2011). Recently, fewer studies have demonstrated that high levels of sLOX‐1 was associated with incidence of acute stroke when compared with age‐ and sex‐ matched controls (Yokota et al., 2016), and may therefore be a predictor of poor functional outcome in patients with large‐artery atherosclerosis stroke (Huang et al., 2017). Other experiments have revealed that sLOX‐1 levels were higher in stroke patients with internal carotid artery stenosis (ICAS) in comparison to those without (Bns, Suwanprasert, & Muengtaweepongsa, 2016). However, the role of sLOX‐1 in acute ischemic stroke (AIS) still remains uncertain. In this study, we aim to further evaluate the relationship between sLOX‐1 levels and the degrees of intracranial artery stenosis and in predicting the long‐term functional outcome in AIS patients.

## 2 MATERIALS AND METHODS

### 2.1 Study population

A total of 272 AIS patients with symptoms within 3 days were consecutively recruited at the First Affiliated Hospital of Wenzhou Medical University from February 2014 to May 2015. All patients were admitted to the stroke unit with diagnosis of AIS according to the World Health Organization Criteria (Stroke, 1989). All participants with the following conditions were excluded: any central nervous system condition such as dementia, Parkinson's disease, subarachnoid hemorrhage or trauma; serious liver or renal insufficiency; heart failure; autoimmune diseases; malignancy; a history of surgery or trauma within recent 3 months.

The consent forms were signed by every patient or their relatives before their inclusion in the study and were also approved by the ethics committee of the First Affiliated Hospital of Wenzhou Medical University.

### 2.2 Data collection

The following patient records were obtained within the first 72 hr on admission: age, gender, medical history of vascular risk factors (smoking, alcohol drinking, hypertension, hyperlipemia, diabetes mellitus, cardiac disease), several biochemical indices, and severity of stroke measured by the National Institute of Health Stroke Scale (NIHSS) (Brott et al., 1989). Functional recovery after one year was assessed by telephone interviews one‐year after stroke onset using the modified Rankin scale (mRS) (Van Swieten, Koudstaal, Visser, Schouten, & Van Gijn, 1988). With reference to other previous study, we defined mild stroke as NIHSS score <5 (Warnecke et al., 2017) and poor functional outcome as mRS score ≥3 (Kim et al., 2012). Furthermore, we used the Trial of Org 10172 in Acute Stroke Treatment criteria, to classify etiology of Ischemic stroke as large‐artery atherosclerosis, cardioembolism, small‐vessel disease, other determined etiology and undetermined etiology (Adams et al., 1993).

### 2.3 Image analysis

All subjects were examined by Magnetic Resonance Imaging (MRI) scans. The size of the infarction area was calculated by diffusion‐weighted imaging (DWI) applying the formula 0.5 * a * b * c (a: the maximal longitudinal diameter; b: the maximal transverse diameter perpendicular to a; c: the number of 5‐mm slices containing the infarct) (Sims et al., 2009). A large infarct volume was defined as larger than 5 cm³. Magnetic resonance angiography (MRA) and Computed tomography angiography (CTA) were performed to evaluate intracranial vascular stenosis or occlusion. We defined percent stenosis of an intracranial artery using the formula: % stenosis = [1 −(D_stenosis_/D_normal_)] × 100,with the normal segment ideally measured from the site distal to the stenotic lesion (Chen et al., 2015). Two independent neuroradiologists measured D_stenosis_ and D_normal_ of stenotic intracranial arteries at different time with deviations between their readings less than 10%. Intracranial arterial stenosis (IAS) in this article, therefore included vertebral artery intracranial segment, basilar artery, internal carotid artery intracranial segment, and the proximal artery of ACA (A1, A2), MCA (M1, M2), PCA (P1, P2).

The degree of IAS was divided into three groups according to the criteria of North American Symptomatic Carotid Endarterectomy Trial (NASCET)(North American Symptomatic Carotid Endarterectomy Trial, 1991): no stenosis group called group 1 (NASCET I: normal), mild to moderate stenosis called group 2 (contain NASCET II to IV ranging from1% to 69% stenosis), and severe stenosis or occlusion called group 3 (contain NASCET V and VI ranging from 70% stenosis to occluded). The location of intracranial arterial stenosis was divided into anterior circulation, posterior circulation, or both. NASCET was used to evaluate the severity of carotid stenosis, although, this method has been widely applied to measure IAS recently (Chen et al., 2015).

### 2.4 Blood collection and laboratory test

Blood samples of all subjects were collected within 24 hr after admission, centrifuged and the aliquots were stored at −80°C for further analysis. The serum sLOX‐1 levels were measured by means of enzyme‐linked immune‐sorbent assays (ELISA). Other parameters including white blood cell count (WBC), neutrophil, total cholesterol (TC), triglycerides (TG), low‐density lipoproteins cholesterol (LDL‐C), high‐density lipoproteins cholesterol (HDL‐C), serum creatinine, blood urea nitrogen (BUN) and uric acid (UA) were also tested in the hospital's biochemistry department.

### 2.5 Statistical analysis

We applied the version 23.0 (SPSS Inc., Chicago, IL) to perform all statistical analyses. Continuous variables were expressed as the mean value ± standard deviation (SD) or medians with inter‐quartile ranges (IQR) according to the normality of data distribution, and performed by Unpaired T‐test or Mann‐Whitney U test. Meanwhile, categorical variables presented as counts and proportions were compared using the Chi‐Square test. The relationship between serum sLOX‐1 levels and long‐term functional outcome in AIS was analyzed by multivariate logistic regression analysis after adjusting for the confounders. The receiver operating characteristic (ROC) curve was utilized to evaluate the accuracy of serum sLOX‐1 to predict the prognosis in AIS and the area under the curve (AUC) was calculated as a measurement of the accuracy of this test. Statistical significance was set at a *p* value <.05.

## 3 RESULTS

### 3.1 Baseline characteristics

A total of 310 patients who met the inclusion criteria were recruited, 301 of them were reachable at 3 months follow‐up, 281 patients at 6 months and 272 patients finished the one‐year follow‐up (15 patients or their relatives refused to offer information and 23 patients were unreachable). The average age of cases (1‐year follow‐up) was 63.04 ± 8.92 years with 96 (35.3%) of patients being women. The median (quartiles) NIHSS score at admission was 4 (IQR 2–5) and an unfavorable outcome at one‐year was found in 71 patients (26.1%). Additional information of AIS patients with favorable or unfavorable long‐term outcomes is described in Table 1. Using the etiological classification of AIS, a significant statistical correlation could be deduced between serum sLOX‐1 and Large and Small‐vessel atherosclerosis disease stroke (2.30 ± 0.82 ng/ml vs. 1.97 ± 0.85 ng/ml, *p* = .011). In addition, a weak but significant connection was found between LDL‐C and sLOX‐1 in stroke people (*p* = .011).

**Table 1:** Baseline characteristics of AIS patients with favorable or unfavorable outcomes

Characteristics	Total (*N *= 272)	The prognosis of one year		*p* value
		Favorable Outcome (*N *= 201)	Unfavorable Outcome (*N *= 71)	
Age (years)	63.04 ± 8.92	62.45 ± 8.65	64.70 ± 9.51	.067
Male no. (%)	176 (64.7)	132 (65.7)	44 (62.0)	NS
SBP (mmHg)	160.43 ± 22.83	160.03 ± 23.63	161.54 ± 23.50	NS
DBP (mmHg)	85.02 ± 12.93	84.90 ± 12.19	85.37 ± 14.91	NS
Hypertension no. (%)	221 (77.6)	160 (79.6)	61 (85.9)	NS
Hyperlipidemia no. (%)	80 (29.4)	59 (29.4)	21 (29.6)	NS
Diabetes no. (%)	91 (33.5)	65 (32.3)	26 (36.6)	NS
Cardiac disease no. (%)	31 (11.4)	20 (10.0)	11 (15.5)	NS
Smoking no. (%)	115 (42.3)	79 (39.3)	36 (50.7)	NS
Alcohol drinking no. (%)	81 (29.8)	63 (31.3)	18 (25.4)	NS
Stroke etiologic subtypes (%)				
Large‐artery atherosclerosis	198 (72.8)	143 (71.1)	55 (77.5)	NS
Cardioembolic	21 (7.7)	16 (8.0)	5 (70.4)	
Small‐vessel disease	48 (17.6)	41 (20.4)	7 (9.9)	
Other or unknown cause	5 (1.8)	1 (0.5)	4 (5.6)	
BMI (kg/m^2^)	23.89 ± 3.01	23.99 ± 3.12	23.62 ± 2.67	NS
Laboratory tests				
WBC (10^9^ /L)	6.52 (5.46–7.71)	6.65 (5.48–7.72)	6.37 (5.39–7.71)	NS
Neutrophils (10^9^ /L)	3.88 (3.19–5.05)	3.81 (3.12–4.88)	3.97 (3.27–5.23)	NS
Lymphocytes (10^9^ /L)	1.81 (1.47–2.32)	1.84 (1.51–2.34)	1.68 (1.32–2.22)	.039
FBG (mmol/L)	5.00 (4.50–6.00)	5.00 (4.50–5.85)	5.10 (4.50–7.10)	NS
HDL‐C (mmol/L)	1.05 (0.92–1.25)	1.05 (0.93–1.27)	1.07 (0.87–1.24)	NS
LDL‐C (mmol/L)	2.88 ± 0.89	2.82 ± 0.84	3.07 ± 0.99	.037
TC (mmol/L)	4.86 ± 1.08	4.80 ± 1.02	5.02 ± 1.26	NS
TG (mmol/L)	1.56 (1.15–2.25)	1.55 (1.14–2.21)	1.67 (1.24–2.38)	NS
SCr (μmol/L)	70.00 (59.00–83.00)	70.00 (58.00–83.00)	70.00 (61.00–85.00)	NS
BUN (mmol/L)	4.70 (3.90–5.60)	4.60 (3.80–5.50)	4.90 (4.20–5.80)	.038
Uric Acid (mmol/L)	301.18 ± 89.98	300.87 ± 84.23	302.07 ± 105.22	NS
sLOX‐1 (ng/ml)	2.26 ± 0.82	2.11 ± 0.80	2.67 ± 0.73	<.0001
Infract volume (cm 3)	1.26 (0.41–3.17)	1.09 (0.40–2.41)	1.83 (0.58–6.20)	.002
NIHSS score on admission, median (IQR)	4.00 (2.00–5.00)	3.00 (2.00–5.00)	5.00 (3.00–8.00)	<.0001
Intracranial arterial stenosis, no. (%)				
No stenosis	75 (27.6)	62 (30.8)	13 (18.3)	.003
Mild to moderate stenosis	160 (58.8)	119 (59.2)	41 (57.7)	
Severe stenosis or occlusion	37 (13.6)	20 (10.0)	17 (23.9)	
Medications, no. (%)				
Statin	230 (84.6)	175 (87.1)	55 (73.3)	.055
Anticoagulation agents	7 (2.6)	4 (2.0)	3 (4.2)	NS
Antiplatelet agents	225 (82.7)	171 (85.1)	54 (76.1)	.085

SBP, systolic blood pressure; DBP, diastolic blood pressure; CAD, coronary artery disease; BMI, body mass index; WBC, leukocyte; FBG, fasting blood glucose; HDL‐C, high‐density cholesterol; LDL‐C, low‐density cholesterol; TC, Total cholesterol; TG, Triglycerides; SCr, serum creatinine; BUN, blood urea nitrogen; sLOX‐1, soluble lectin‐like oxidized low density lipoprotein receptor‐1; IQR, interquartile range; NIHSS, National Institutes of Health Stroke Scale.

### 3.2 The stroke severity and the cerebral infarction volume

AIS patients were divided into two groups based on the NIHSS score on admission: 178 patients were with mild stroke (NIHSS score <5) and 94 patients presented with moderate stroke (NIHSS score ≥5). There were positive correlations between the sLOX‐1 levels and the NIHSS score (*r* = .152, *p* = .012). It turns out to be that serum sLOX‐1 levels were significantly higher in the moderate stroke patients compared to the mild stroke group (2.43 ± 0.75 ng/ml vs. 2.16 ± 0.84 ng/ml, *p* = .011).

All patients have gone through brain MRI scans. Large infarct volume (the volume ≥5 cm³) was found in 44 patients (16.2%). There was no significant difference in the level of serum sLOX‐1 between the small and large infarct volume groups (2.25 ± 0.83 ng/ml vs. 2.28 ± 0.77 ng/ml, *p* > .05).

### 3.3 sLOX‐1 and the degrees of intracranial artery stenosis

In this study, 75 patients (27.6%) were found without stenosis in the normal group, 156 patients (58.8%) had mild to moderate stenosis (ranged from1% to 69% stenosis) and 36 patients (13.6%) were with severe stenosis or occlusion (ranged from 70% stenosis to occluded). The difference between sLOX‐1 concentration and the severity of IAS was statistically significant between groups (*p* = .029). The levels of serum sLOX‐1 in severe stenosis group were significantly higher compared to the normal (2.59 ± 0.86 ng/ml vs. 2.18 ± 0.68 ng/ml, *p* = .015) and mild to moderate stenosis group (2.59 ± 0.86 ng/ml vs. 2.22 ± 0.85 ng/ml, *p* = .019), while the biomarker has no statistical significance between the mild to moderate stenosis group compared with normal group (*p* > .05). In addition, the relationship between sLOX‐1 levels and the locations of IAS is shown on Table 2.

**Table 2:** sLOX‐1 levels in different degree of IAS

Locations	Total M ± SD, ng/ml	Group 1	Group 2	Group 3	*p*
Anterior circulation	2.31 ± 0.86	—	2.28 ± 0.83	2.57 ± 1.10	NS
Posterior circulation	2.30 ± 0.90	—	2.25 ± 0.90	2.58 ± 0.91	NS
Both circulations	2.27 ± 0.86	—	2.16 ± 0.86	2.59 ± 0.83	.031
Total	2.26 ± 0.82	2.18 ± 0.68^a^	2.22 ± 0.85^b^^,^ c	2.59 ± 0.86	.029

a *p* = .015, Severe stenosis group compared with normal group.

b *p* = .019, Severe stenosis group compared with mild to moderate stenosis group.

c *p* = .720, Mild to moderate stenosis group compared with normal group.

### 3.4 sLOX‐1 and one‐year functional outcome

Out of the 272 patients who completed the one‐year follow‐up, an unfavorable outcome was found in 113 patients (at 3 months), 68 patients (at 6 months) and 71 patients (in 1 year). There was a significant difference in sLOX‐1 levels between patients with good prognosis and those with poor prognosis at 3 months (2.09 ± 0.80 ng/ml vs. 2.49 ± 0.79 ng/ml, *p* < .001). There was however a weaker correlation between sLOX‐1 levels and prognosis at 6 months (2.20 ± 0.84 ng/ml vs. 2.43 ± 0.73 ng/ml, *p* = .041). We also found that the serum sLOX‐1 levels were observably lower in patients with favorable functional outcome in comparison to those with poor functional outcome (2.11 ± 0.80 ng/ml vs. 2.67 ± 0.73 ng/ml, *p* < .001; Table 1). In univariate logistic regression analysis, the concentration of serum sLOX‐1 was associated with a poor long‐term outcome in AIS patients with an unadjusted OR of 2.441 (95% CI, 1.695–3.571, *p* < .001). After adjusting for all potential confounders (age, gender, hypertension, hyperlipidemia, diabetes mellitus, cardiac disease, smoking, alcohol drinking, the NIHSS scores on admission, the degrees of AIS, infract volume, stroke etiologic subtypes, neutrophils, lymphocytes, LDL, BUN, creatinine, the use of antiplatelet agents, statin and anticoagulation agents), sLOX‐1 was still an independent predictor for functional outcome in stroke cases with an adjusted OR of 2.946 (95% CI, 1.788–4.856, *p* < .001). Furthermore, the degrees of IAS were closely related to long‐term prognosis of AIS with an adjusted OR of 1.912 (95% CI, 1.052–3.477, *p* = .034). In addition, NIHSS scores, smoking and IAS were also independent predictors for functional outcome in patients with AIS (Table 3).

**Table 3:** Logistic regression model with predictors of unfavorable outcome (*n *= 272)

Characteristics	Unadjusted OR (95% CI)	*p* value	Adjusted OR (95% CI)	*p* value
Age (years)	1.030 (0.998–1.062)	.068	—	—
Male no. (%)	0.852 (0.486–1.492)	.575	—	—
SBP (mmHg)	1.003 (0.991–1.015)	.634	—	—
DBP (mmHg)	1.003 (0.982–1.024)	.792	—	—
Hypertension no. (%)	1.563 (0.737–3.314)	.244	—	—
Hyperlipidemia no. (%)	1.011 (0.559–1.829)	.972	—	—
Diabetes no. (%)	1.209 (0.686–2.129)	.511	—	—
Cardiac disease no. (%)	1.659 (0.752–3.661)	.210	—	—
Smoking no. (%)	1.588 (0.921–2.738)	.098	4.942 (1.636–5.927)	.005
Alcohol drinking no. (%)	0.744 (0.403–1.372)	.344	—	—
Stroke etiologic subtypes (%)	0.909 (0.654–1.265)	.572	1.542 (0.932–2.551)	.092
Large‐artery atherosclerosis	—	—	—	—
Cardioembolic	—	—	—	—
Small‐vessel disease	—	—	—	—
Other or unknown cause	—	—	—	—
BMI (kg/m^2^)	0.959 (0.875–1.051)	.373	—	—
Laboratory tests			—	—
WBC (10.9/L)	1.057 (0.915–1.222)	.451	—	—
Neutrophils (10.9/L)	1.163 (0.992–1.362)	.062	—	—
Lymphocytes (10.9/L)	0.595 (0.367–0.965)	.036	0.434 (0.210–0.900)	.025
FBG (mmol/L)	1.134 (1.017–1.265)	.024	—	—
HDL‐C (mmol/L)	0.659 (0.247–1.757)	.405	—	—
LDL‐C (mmol/L)	1.377 (1.018–1.865)	.038	—	—
TC (mmol/L)	1.200 (0.936–1.539)	.150	—	—
TG (mmol/L)	0.934 (0.729–1.198)	.592	—	—
SCr (μmol/L)	1.010 (0.998–1.021)	.106	—	—
BUN (mmol/L)	1.238 (1.034–1.482)	.020	—	—
Uric acid (mmol/L)	1.000 (0.997–1.003)	.923	—	—
sLOX‐1 (ng/ml)	2.441 (1.695–3.571)	<.001	2.946 (1.788–4.856)	<.001
Infract volume (cm^3^)	1.028 (1.000–1.057)	.049	—	—
NIHSS score on admission, median (IQR)	1.381 (1.240–1.538)	<.001	1.453 (1.248–1.691)	<.001
Intracranial arterial stenosis, no. (%)	1.998 (1.274–3.133)	.003	1.912 (1.052–3.477)	.034
No stenosis	—	—	—	—
Mild to moderate stenosis	—	—	—	—
Severe stenosis or occlusion	—	—	—	—
Medications, no. (%)				
Antiplatelet agents	0.511 (0.256–1.021)	.057	—	—
Anticoagulation agents	2.173 (0.474–9.956)	.318	—	—
Statin	0.557 (0.285–1.088)	.087	—	—

OR, odds ratio; CI, confidence interval; IQR, interquartile range; SBP, systolic blood pressure; DBP, diastolic blood pressure; CAD, coronary artery disease; BMI, body mass index; WBC, leukocyte; FBG, fasting blood glucose; HDL‐C, high‐density cholesterol; LDL‐C, low‐density cholesterol; TC, Total cholesterol; TG, Triglycerides; SCr, serum creatinine; BUN, blood urea nitrogen; sLOX‐1, soluble lectin‐like oxidized low density lipoprotein receptor‐1; NIHSS, National Institutes of Health Stroke Scale.

**Figure 1:**
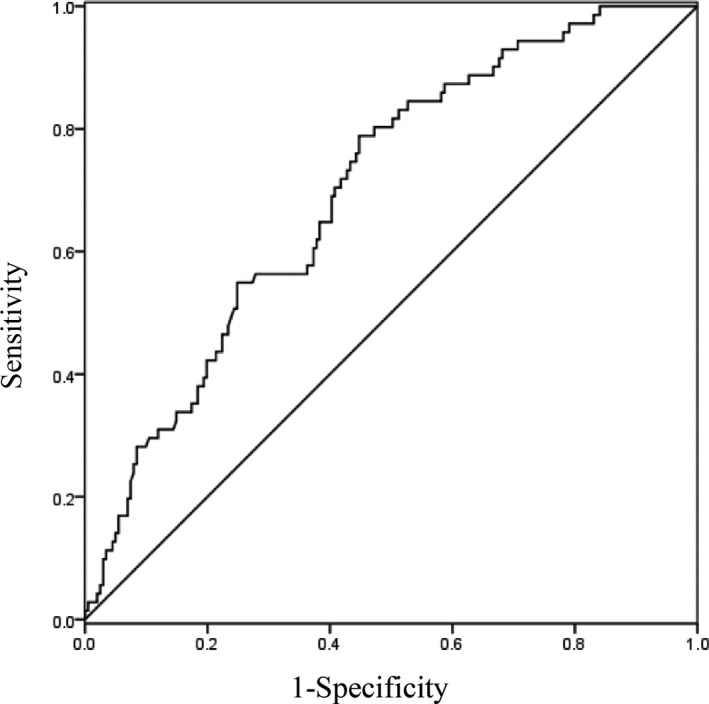
ROC curve of sLOX‐1 for predicting one‐year outcome in acute ischemic stroke

**Figure 2:**
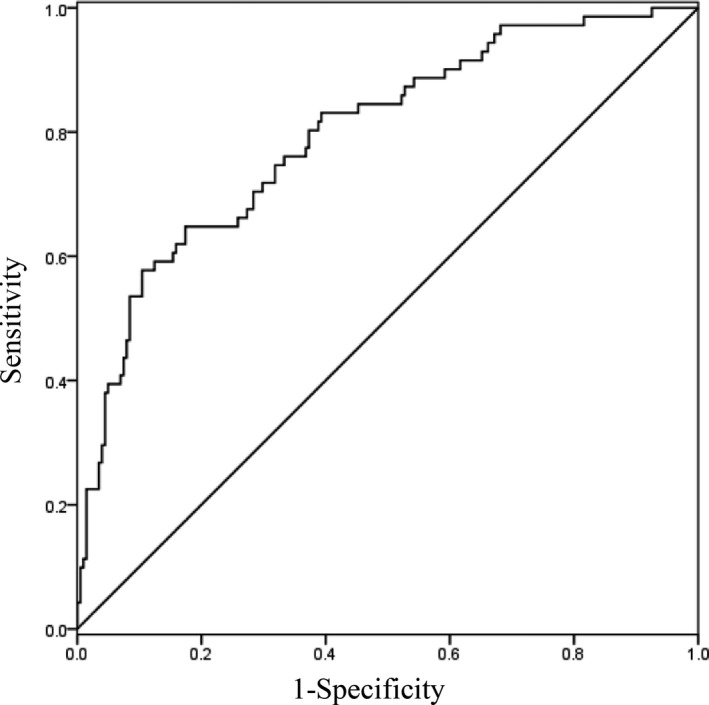
The combination ROC curve of sLOX‐1 and NIHSS for predicting one‐year outcome in acute ischemic stroke

Based on the receiver operating characteristic (ROC) curve analysis, the optimal cutoff value of serum sLOX‐1 for predicting one‐year prognosis was projected to be 2.077 ng/ml with a sensitivity of 78.9% and a specificity of 55.2% (area under the curve: 0.700, 95% CI (0.633–0.766; *p* < .001). The ROC curves of sLOX‐1 levels for prediction of one‐year functional outcome is shown in Figure 1. The combination of serum sLOX‐1 and the NIHSS scores showed greater accuracy (AUC, 0.796; 95% CI, 0.735–0.857; *p* < .0001) than the NIHSS score or sLOX‐1 levels alone (Figure 2).

## 4 DISCUSSION

From our study results, serum sLOX‐1 may play a negative role in the recovery of cerebral infarction. We observed that the expression of serum sLOX‐1 was increased significantly in patients with undesired long‐term outcome (*p* < .001). To the best of our knowledge this was the first study which investigated the relationship between sLOX‐1 and the long‐term prognosis of AIS. Furthermore, our results indicated statistically significant correlation between the concentration of sLOX‐1 in serum and the degrees of IAS (*p* = .029). We therefore speculate that sLOX‐1 may be a potential predictor for IAS.

LOX‐1 is the main receptor of Ox‐LDL in endothelial cells. Through the combination with Ox‐LDL, LOX‐1 may cause lots of endothelial dysfunction and would contribute to vascular atherosclerosis, inflammation, acute coronary syndrome and stroke (Chen et al., 2003; Honjo et al., 2003; Inoue, Arai, Kurihara, Kita, & Sawamura, 2005; Inoue et al., 2010). In addition, the vulnerability of atherosclerotic plaques could be reflected by amount of LOX‐1 to some extent (Kataoka et al., 2001). Many studies have indicated that soluble lectin‐like oxidized low‐density lipoprotein receptor‐1 (sLOX‐1), could be measured to reflect the expression of LOX‐1 (Brinkley et al., 2008; Murase et al., 2000).

Hayashida et al. (2005) found that increased level of sLOX‐1 could reflect the vulnerability of plaque, since elevated levels of soluble receptors might weaken fibrous caps, which would lead to plaque disruption, eventually causing acute coronary syndrome. Kobayashi et al. (2013) further verified this assertion concluding with similar findings. Many previous studies have confirmed the relationship between sLOX‐1 levels and cardiovascular disease. Lubrano, Del Turco, Nicolini, Di Cecco, and Basta (2008) found that serum sLOX‐1 was up‐regulated during the progression of coronary artery disease and is related to the severity of cardiovascular disease, and in some extent, the serum sLOX‐1 levels could indicate the progression of coronary artery disease. Another study demonstrated that increased sLOX‐1 levels made the patients with acute coronary syndrome more vulnerable to disease recurrence and death (Kume, Mitsuoka, Hayashida, Tanaka, & Kita, 2010). In addition, Li et al. (2011) suggested that post‐procedural sLOX‐1 levels might be useful to assess the risk of coronary stent re‐stenosis and the degree of lumen loss in patients with stable coronary artery disease undergoing percutaneous coronary interventional therapy. A recent research showed that circulating sLOX‐1 levels were increased markedly in patients with coronary slow flow phenomenon with high expression of the soluble receptors having considerable influence in pathogenesis of coronary slow flow phenomenon (Caglar et al., 2016). Serum sLOX‐1 also has a pronounce effect on stroke progression according to previous researches. Yokota et al. (2016) revealed that sLOX‐1 level was elevated in stroke patients compared to gender and age matched controls. In the present study, obvious increase in circulating sLOX‐1 was noticed in patients with AIS compared with those in control group. Furthermore, Huang et al. (2017). found that sLOX‐1 were detected lower in healthy controls and closely related to the short‐term outcome in subjects with large‐artery atherosclerosis stroke. Consistently, our study found that the higher sLOX‐1 levels strongly correlated with a more serious stroke status and poorer long‐term functional outcome.

Based on previous population or hospital prospective studies and the large randomized trials, it has been established that carotid plaques (European Carotid Surgery Trialists’ Collaborative Group, 1991; North American Symptomatic Carotid Endarterectomy Trial C, 1991) and IAS (Sacco, Kargman, Gu, & Zamanillo, 1995; Wityk et al., 1996) were the major causes of stroke. There are however differences between the two especially with regards to risk factors and epidemiology. Wbo‐Keun Seo et al. (Kim et al., 2010; Wityk et al., 1996) had found that IAS often occurs predominantly in Asians, Blacks, and Spanish, while Europeans and Americans more possibly suffer from stenosis of extra cranial arteries. Several previous studies speculated that IAS was more likely to happen in Asians (De Silva et al., 2007). Liu, Tu, Yip, and Su (1996) reported in their study that the Chinese had the probability of developing a much more serious intracranial vessel lesion. This hypothesis was later confirmed by Cui, Wu, Zeng, Xiao, and Liu (2015). Consistently, our results indicated that IAS was significantly associated with the prognosis of AIS with an OR of 1.998 (95% CI, 1.274–3.133, *p* = .003). Intracranial artery stenosis, which has been speculated to be the pathological foundation and pathogenesis of cerebral infarction, is mainly caused by primary atherosclerosis (Carvalho et al., 2014). Previous study had found that higher serum sLOX‐1 levels were detected in stroke patients with internal carotid artery stenosis (ICAS) as compared to those without (Bns et al., 2016). The exact relationship between sLOX‐1 levels and IAS however is still uncertain. In this study, we observed that the expression of sLOX‐1 levels correlated remarkably to the severity of IAS (Table 2).

Several studies have confirmed that LDL‐C and its oxidized form was an independent risk factor of stroke (Cholesterol Treatment Trialists’ (CTT) Collaboration, 2015; Wang et al., 2017). In our study, LDL‐C weakly correlated with the outcome of AIS (*p* = .038) with an unadjusted OR of 1.377 (95% CI, 1.018–1.865), while, serum sLOX‐1 showed strong relation with clinical prognosis of AIS (*p* < .0001) with an adjusted OR of 2.946 (95% CI, 1.788–4.856). Thus, sLOX‐1 may be a more sensitive predictor than LDL‐C for stroke outcome. Furthermore, it has been reported that LOX‐1 might be an original therapeutic target in atherosclerotic diseases when applying statins and antioxidant properties (Muscoli et al., 2004). The circulating sLOX‐1, soluble form of LOX‐1, could be tested to reflect the effect of the novel therapy. However, all of these hypotheses need further investigation.

Our study encountered some limitations that should be taken into consideration. Firstly, this is a single centered study as such the probability of selection bias is higher. With regards to, measuring serum sLOX‐1 levels, our study did not put into account the analyses of circulating sLOX‐1 at different stages of AIS. We are therefore of the view that dynamic monitoring of change in sLOX‐1 levels at different stages need to be studied further. Rehabilitation still remains a crucial part of functional recovery after stroke. Our study however did not establish the rehabilitative training situations of patients after they were discharged. Finally, we used a comparatively small sample size with majority of subjects having mild condition which we believe could influence the result. In further study, the population size should be enlarged to reduce the effect of random variations.

## 5 CONCLUSION

In conclusion, the present study suggests that sLOX‐1 could be an available indicator for predicting the degrees of IAS and the long‐term functional outcome in stroke patients. If these conclusions could be confirmed by further studies, sLOX‐1 may become a promising biomarker for diagnosing and predicting AIS at early stage and at the same time could probably become an important means of evaluating the degrees of intracranial artery stenosis before imaging.

## CONFLICT OF INTEREST

None declared.
